# Supplementary figures and images for: Eukaryotic gut community of the bat Myotis arescens in anthropized landscapes in Chile
Source: PeerJ. 2025 Jun 30;13:e19563. doi: 10.7717/peerj.19563 (PMC12225633; doi:10.7717/peerj.19563)

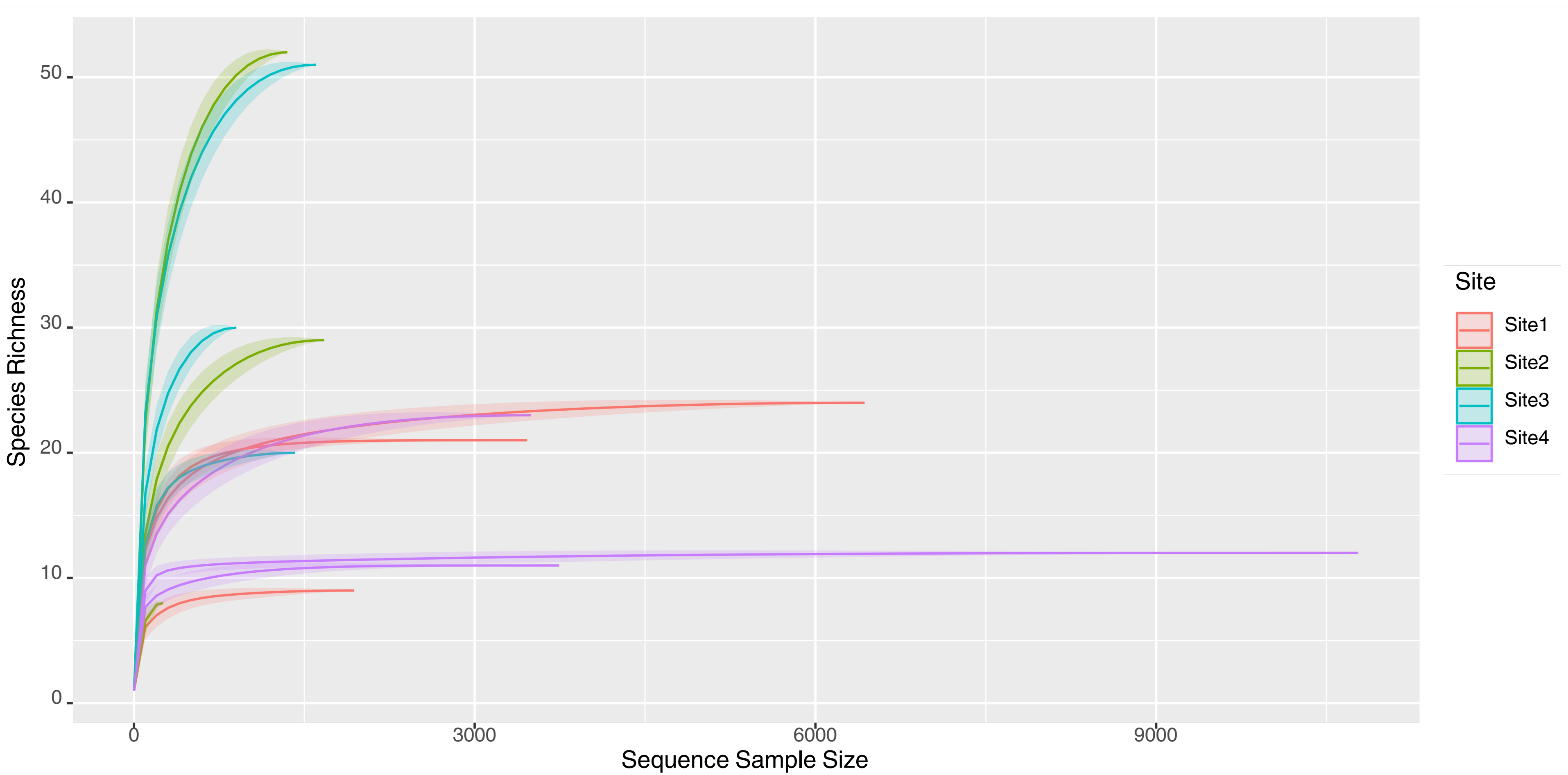

Supplement: Supplemental Information 1 [file peerj-13-19563-s001.pdf]

NMDS based on Jaccard distance

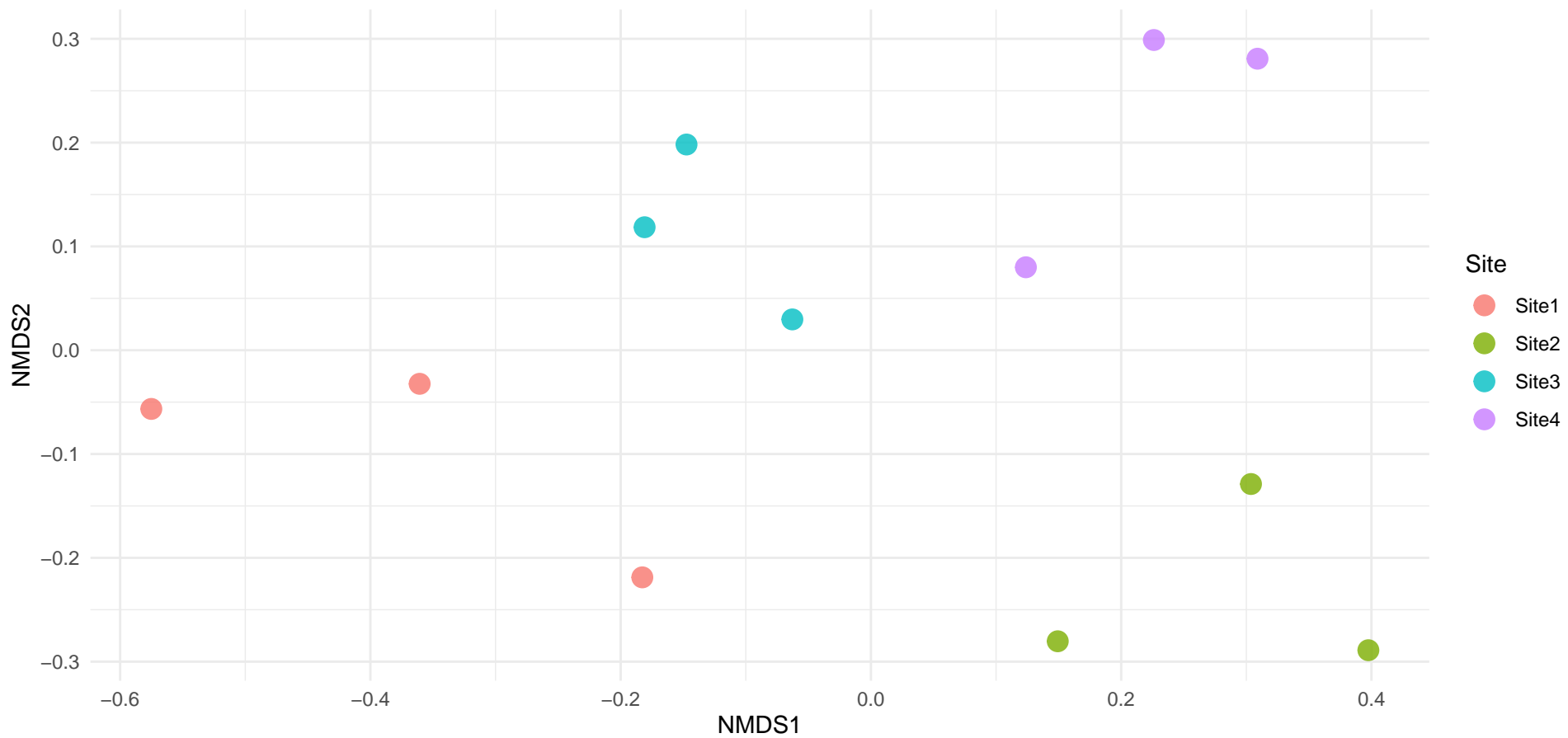

Supplement: Supplemental Information 2 [file peerj-13-19563-s002.pdf]

PCA Biplot of Site Characteristics

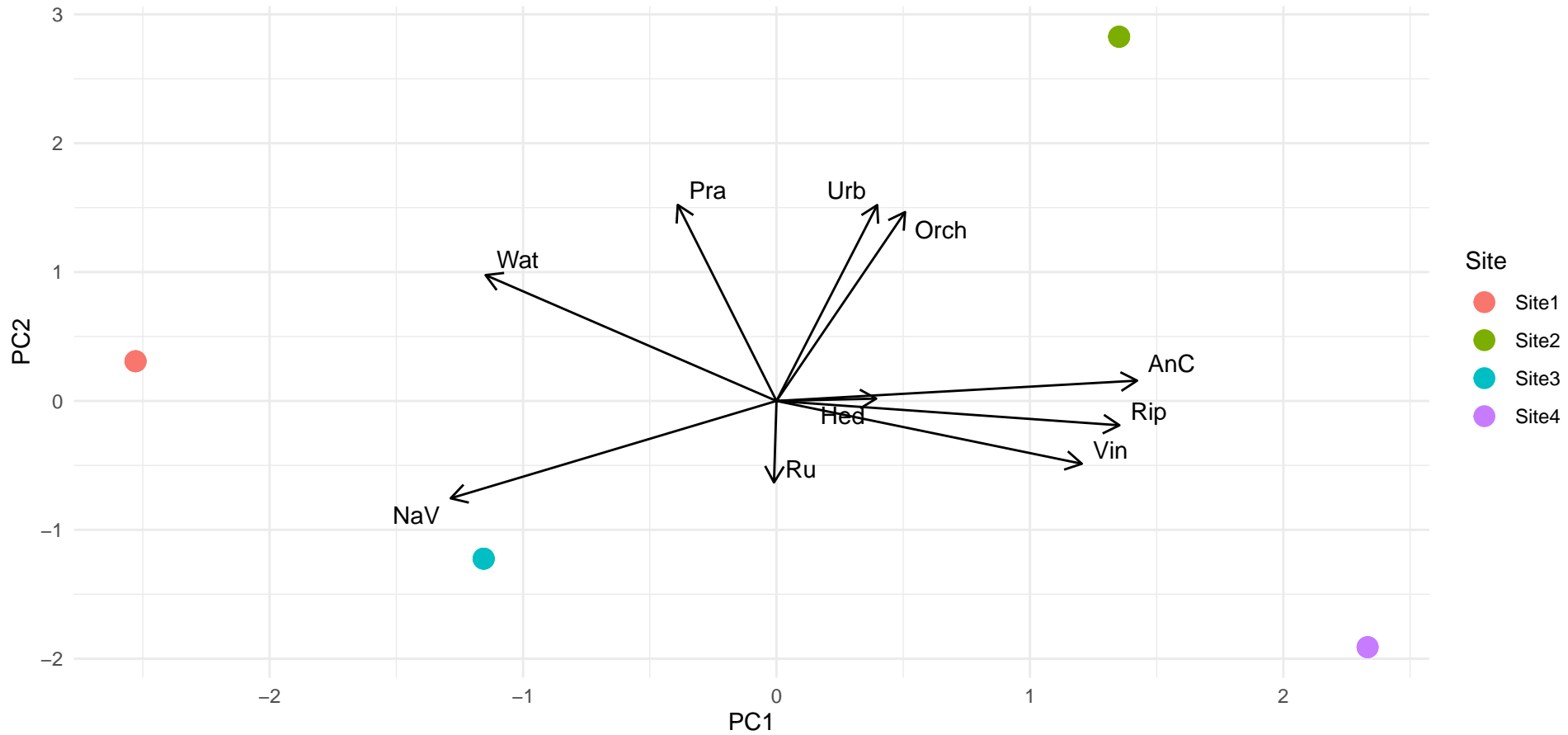

Supplement: Supplemental Information 3 — Points represent sampling sites, colored by site ID. Arrows indicate the direction and strength of environmental variables contributing to the ordination space. Sites 2 and 4 are characterized by high levels of anthropogenic cover (AnC), vineyards (Vin), and riparian vegetation (Rip), while Site 3 aligns more with natural vegetation (NaV) and rural areas (Ru). [file peerj-13-19563-s003.pdf]

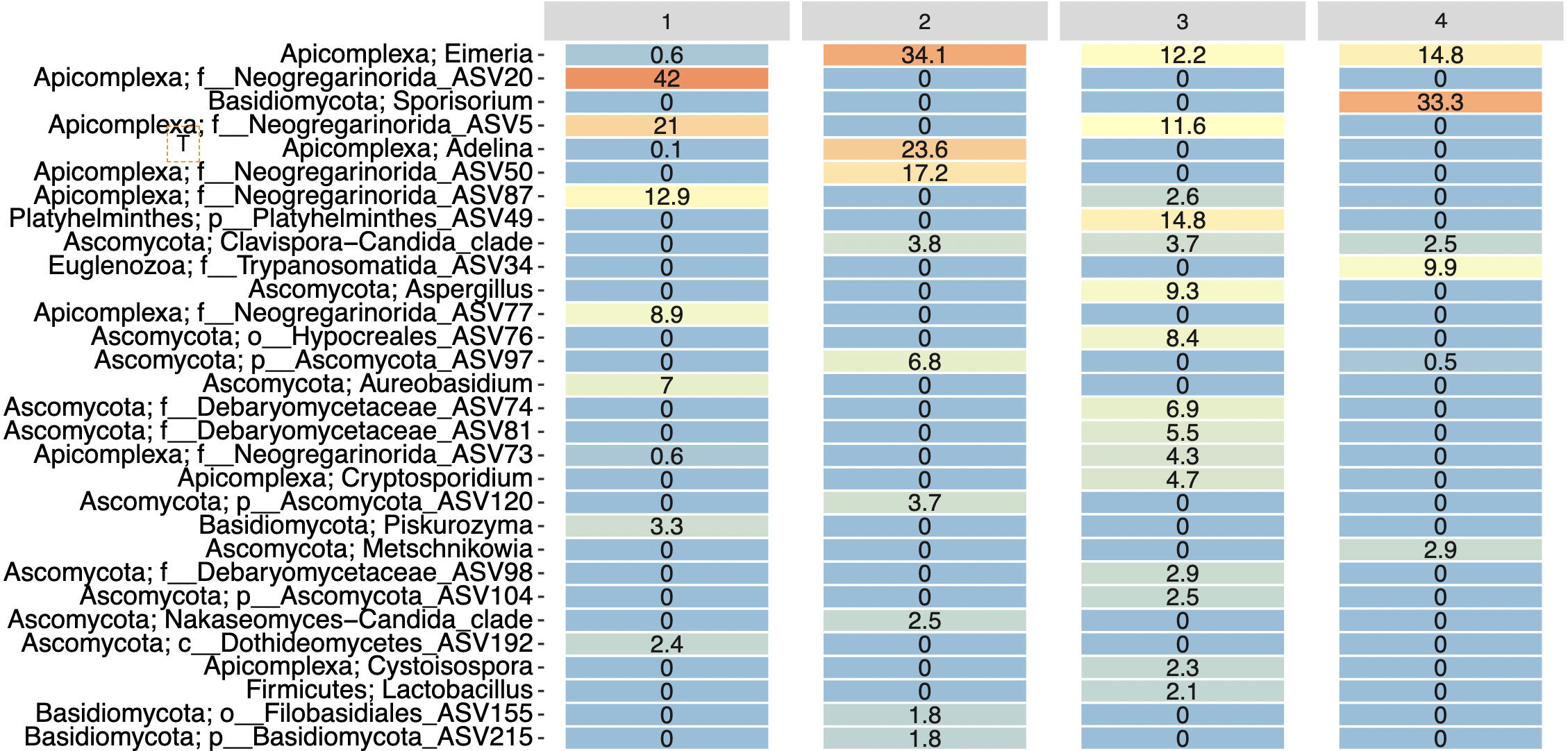

Supplement: Supplemental Information 4 — Heatmap of relative abundance of the 30 most abundant ASV from each site. The numbers indicate the percentage of ASV abundance in each sample site. Orange represents the highest percentage and blue the lower percentage. [file peerj-13-19563-s004.png]
